# From Nature to Nurture: The Science and Applications of Snail Slime in Health and Beauty

**DOI:** 10.1111/jocd.70002

**Published:** 2025-02-19

**Authors:** Muhammad Rashad, Simone Sampò, Amelia Cataldi, Susi Zara

**Affiliations:** ^1^ Department of Pharmacy “G. d'Annunzio” University of Chieti‐Pescara Chieti Italy; ^2^ International Institution of Heliciculture of Cherasco–Lumacheria Italiana Srl Cherasco Italy

**Keywords:** biomolecules, cosmetics, cruelty‐free extraction, human health, snail slime, sustainability

## Abstract

**Background:**

Snail slime (SS), a complex biological substance produced by various snail species, has garnered significant attention in recent years due to its diverse applications in health, cosmetics, and biotechnology.

**Aims:**

Our previous review focused on the biological activities of SS, while the current one explores the science behind SS with a special focus on environmental factors affecting its quality and quantity, non‐lethal extraction methods, its composition, current applications in health and cosmetics followed by its emerging applications, and future prospects while achieving sustainability.

**Methods:**

A literature review on background, uses in health and cosmetics, and future prospects of SS was conducted. PubMed and Google Scholar were used to find the key articles exploring SS and the data is summarized and described here.

**Results:**

Extraction methods range from traditional farming practices to advanced, non‐invasive techniques aimed at minimizing stress on snails. Emerging applications include potential use in sustained and targeted drug delivery systems, tissue engineering, and as components in advanced biomaterials. Future perspectives involve technological advancements in production, such as precision farming and biotechnology‐enhanced mucin production. The development of synthetic alternatives and sustainable practices is crucial for the industry's long‐term viability.

**Conclusion:**

As research continues to uncover new properties and applications, SS is poised to play an increasingly important role in the health and beauty industries, highlighting the need for balanced exploitation, standardization, quality control, and regulatory compliance in its production and use.

## 1 Introduction

Snails, slow‐moving mollusks with spiral shells, have captivated human interest for centuries. Found in diverse habitats from gardens to oceans, these remarkable creatures have adapted to a wide range of environments. While often seen as garden pests, snails play crucial roles in ecosystems as decomposers and food sources for various animals. Their unique anatomy, featuring a muscular foot for locomotion and a protective shell, has evolved over millions of years [1, 2]. Beyond their ecological importance, snails have found their way into human culture, appearing in cuisine, art, and even health and skincare. Their seemingly simple existence belies a complex biology that continues to intrigue scientists and nature enthusiasts alike.

Snail slime (SS), a natural mucus secreted by snails for locomotion and protection, scientifically known as snail mucin or snail serum, has emerged as an unlikely star in the health and beauty world. SS has gained significant traction in industries dedicated to health and beauty products due to potential benefits [3].

In skin care, snail mucin is most commonly found in serums, essences, and moisturizers. These products often claim to address multiple skin concerns simultaneously, including fine lines, uneven texture, dullness, and dehydration. Some users reported improved skin healing, reduced acne scarring, and a more radiant complexion with regular use [3]. Others reported improvements in skin texture, reduction of fine lines, and enhanced overall skin health with consistent use. The popularity of snail mucin products has particularly soared in Korean and Japanese skincare routines, which have significantly influenced global beauty trends. Its ability to hydrate, repair, and rejuvenate skin has made it a staple in anti‐aging formulations [4].

Beyond cosmetics, SS shows promise in medical applications. Some studies suggest that it can accelerate wound healing and reduce scarring, potentially due to its growth factor content and antimicrobial properties [5]. This has led to interest in its use for treating minor cuts, burns, and even certain skin conditions like eczema and rosacea [6]. However, it is important to note that while anecdotal evidence is abundant, rigorous clinical studies on these applications are still limited.

Moreover, the extraction of snail mucin for beauty products has raised ethical questions. Traditional methods involve stressing the snails to produce mucin, but many companies are now using more ethical‐approved techniques. These techniques include allowing snails to move over mesh in a calm environment and collecting the mucin they naturally produce. The development of these “cruelty‐free” extraction methods [7] has been crucial in making snail mucin products more palatable to ethically conscious consumers.

As the popularity of snail mucin in health and beauty products continues to grow, so does the need for more comprehensive research. The scientific community is still working to fully understand its mechanisms of action and long‐term effects on skin health. Future studies may help to optimize its use in both cosmetic and medical applications, potentially opening new avenues for the use of snail mucin in dermatology and health care. This review explores the science behind SS with a special focus on environmental factors affecting its quality and quantity, non‐lethal extraction methods, composition, current applications in health and cosmetics followed by emerging applications, and future prospects while achieving sustainability.

## 2 Science of Snail Slime

The science of SS is a fascinating area of study that has gained significant attention in recent years. This unique substance, produced by various species of snails, has intrigued researchers and beauty enthusiasts alike due to its complex composition and potential beneficial properties. Understanding the science behind SS involves exploring its composition, the biological process of production, and the factors that influence its quality. At its core, SS is a complex mixture of compounds that serve multiple purposes for the snail, primarily in locomotion and protection [2]. The composition of this mucus is what makes it particularly interesting for potential applications in health and beauty.

The biological process of SS production is equally intriguing. Snails produce different types of mucus through specialized glands in their body. The pedal glands, located in the snail's foot, are responsible for producing the slime used for locomotion [8]. This slime helps the snail move smoothly across surfaces while also providing a protective barrier between the snail's body and the ground. The mucus secreted by pedal glands contains more than 99% water, glycosaminoglycans (GAGs) and some minerals that help the animal in locomotion and to stick to a site via this gel‐like secretion. According to Greistorfer et al. only three bands of proteins (82, 97, 175 kDa) were detected in *Helix aspersa* during proteomics analysis [9] emphasizing that mucus obtained from pedal glands has the least importance in burns and cosmetics.

Another type of mucus is produced by the mantle glands, located near the snail's shell, helping to shield the snail from potential harm and aiding in shell formation and repair [9]. The mucus secreted by the mantle glands contains the major proteins, GAGs, mucopolysaccharides, minerals and other important enzymes useful in burns, wound healing and cosmetics [9]. The process of slime production involves the secretion of mucus precursors from these gland cells. When exposed to water, these precursors rapidly swell and hydrate, forming a network of interlinked polymers that give the slime its characteristic viscous texture [10].

The quality and composition of SS can be influenced by various factors. The species of snail is a primary determinant, as different species may produce slime with varying compositions. For instance, the mucus from *H. aspersa* (garden snail) might differ in its component ratios from that of *Achatina fulica* (giant African land snail) [11].

Diet plays a crucial role in the quality of SS. The nutritional intake of the snail can affect the components present in the slime. Snails fed on a diet rich in certain nutrients (i.e., calcium) may produce slime with a different composition compared to those on a less varied diet [12]. This factor is particularly important in the commercial production of SS, where snail diets may be carefully controlled to optimize the desire components in the mucus.

Environmental factors also play a significant role in slime production and quality. Humidity, temperature, and stress levels can influence the quantity and composition of the slime produced. Snails in more stressful environments may produce slime with different properties compared to those in more favorable conditions [13]. This understanding has led to the development of specialized snail farms designed to create optimal conditions for high‐quality slime production.

The age of the snail is another crucial factor that can impact slime quality. Younger snails may produce more slime with a better composition compared to older ones. Similarly, seasonal changes can affect a snail's metabolism and, consequently, its slime production [14]. These factors add to the complexity of consistently producing high‐quality SS for commercial purposes.

The science of SS is a complex and fascinating field that intersects biology, chemistry, and dermatology. The unique composition of this natural substance, with its blend of potentially beneficial components, has sparked significant interest in its applications for skin health and beauty. As research continues, our understanding of SS's properties and potential uses is likely to expand, opening up new avenues for its application in both cosmetic and medical fields.

## 3 Extraction and Production

SS extraction and production have gained significant attention in recent years due to the increasing demand for snail mucin in cosmetics and pharmaceuticals. This process involves several stages, from snail farming to extraction and processing, each with its own set of practices and ethical considerations.

In heliciculture or snail farming, the foundation of SS production, the most commonly farmed species for mucin production is *H. aspersa* . According to Cobbinah et al. successful snail farming requires careful control of environmental factors such as temperature (20°C–25°C), humidity (75%–95%), and light exposure [15, 16]. Snails are typically housed in enclosed systems with soil‐filled pens or plastic containers. Their diet consists of calcium‐rich vegetables and specially formulated feeds to ensure optimal health and mucin production. Proper sanitation and disease management are crucial to maintain a healthy snail population [17].

**FIGURE 1:**
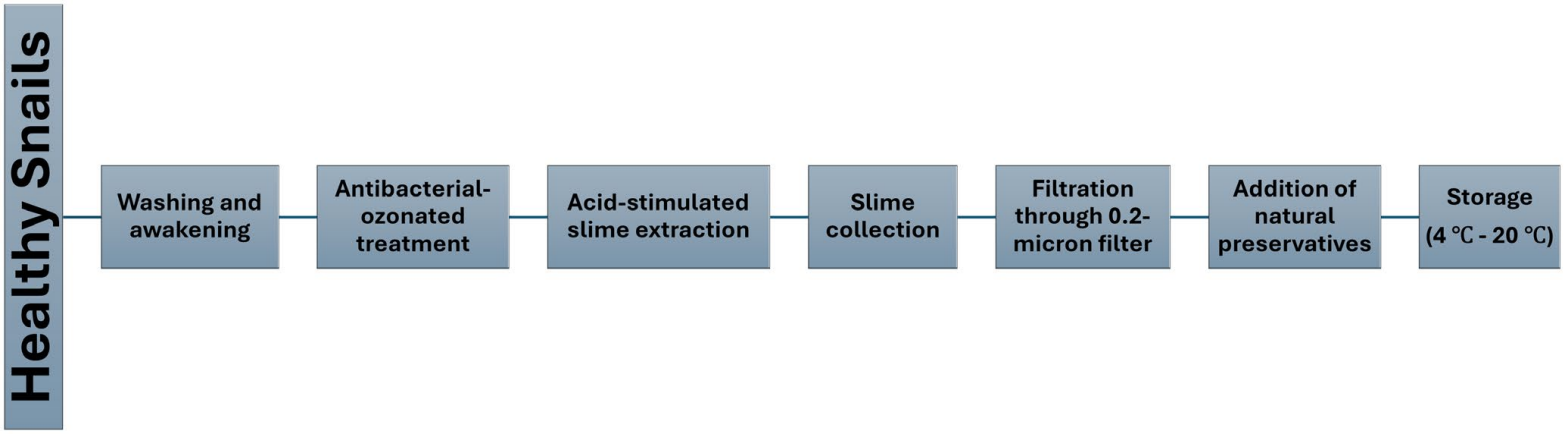
A flowchart describing the extraction and production of SS using non‐lethal Muller method.

**FIGURE 2:**
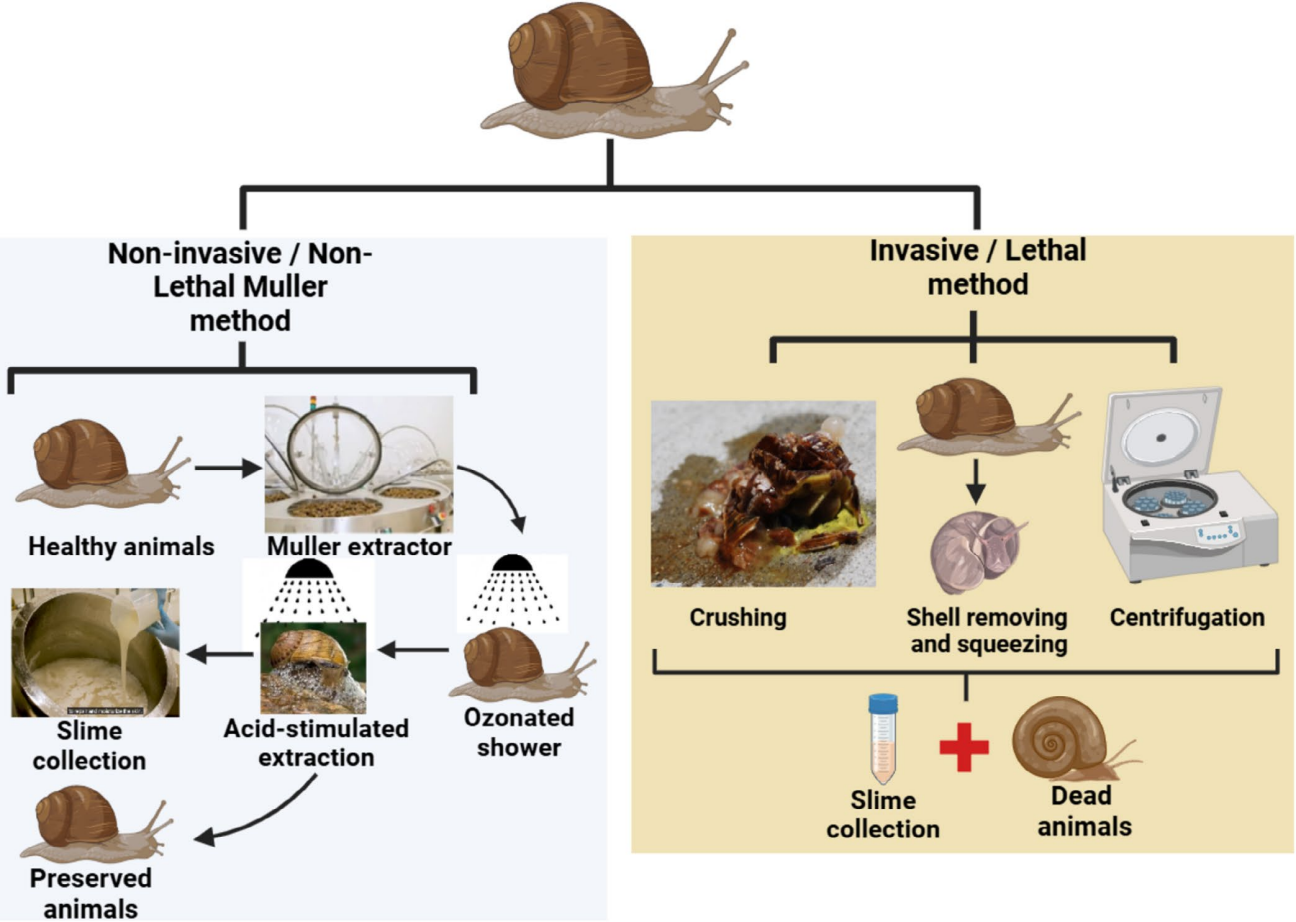
Comparative illustration of non‐lethal SS Muller extraction and production method (left panel) and lethal (right panel) SS extraction and production method.

There are several methods for extracting snail mucin, ranging from traditional to more modern techniques. Non‐lethal methods include stimulated production, where snails are induced to produce mucin through acid stimulation [18] (Figure 1). Unfortunately, some lethal methods are still in use, such as crushing whole snails or using solvent extraction, mechanical stimulation, or centrifugation [3] which raises significant ethical concerns (Figure 2).

After extraction, the raw mucin undergoes several processing steps. These typically include filtration to remove debris and impurities, centrifugation to separate different components of the mucin, lyophilization (freeze‐drying) to produce a stable powder form, and sterilization to ensure product safety for cosmetic or pharmaceutical use. Advanced techniques such as ultrafiltration and chromatography may be employed to isolate specific bioactive compounds from the mucin [6].

Implementing sustainable practices is essential for the long‐term viability of the industry and to minimize environmental impact. This includes optimizing farming conditions to reduce the use of resources, developing more efficient and humane extraction methods, and exploring ways to utilize by‐products of the snail farming [19]. The extraction and production of SS raises several ethical concerns. Animal welfare is a primary consideration, with non‐lethal extraction methods preferred to minimize harm to snails [20]. Environmental impact is another concern, as snail farming, if not properly managed, could potentially lead to the introduction of non‐native species into local ecosystems. Therefore, sustainable farming practices are crucial to mitigate this risk [21].

There is a growing demand for transparency in the cosmetics industry regarding the sourcing and production methods of snail mucin. Some companies (i.e., Lumacheria Italiana srl.) have developed innovative “cruelty‐free” Cherasco method for their snail mucin products [2, 22]. Additionally, the production of snail mucin for cosmetic or pharmaceutical use must comply with relevant regulations and safety standards. This includes ensuring that extraction and processing methods do not introduce harmful contaminants [23].

## 4 Composition of Snail Slime

SS, a complex bioactive substance, is primarily composed of water (90%–99.7%) and a diverse array of compounds. The dry weight typically includes 5%–9% proteins, 3%–5% GAGs, and 1.3%–1.6% mucopolysaccharides. Key components include hyaluronic acid (HA) (< 1 mg/g), glycolic acid (GA) (up to 4%), and allantoin (0.3%–0.5%). The slime also contains collagen, elastin, and various glycoprotein enzymes [9], each contributing to its purported skincare benefits. GA acts as a gentle exfoliant, promoting cell turnover and improving skin texture [24]. HA is a powerful humectant capable of holding up to 1000 times water of its weight, drawing moisture to the skin and helping maintain hydration [25]. This substance helps to hydrate the skin deeply, plumping it up and reducing the appearance of fine lines. The presence of HA contributes to the moisturizing properties often associated with SS‐based products. Allantoin is known for its soothing and healing properties, while the proteins and peptides in the mucin may support collagen production and skin elasticity. Collagen and elastin, both proteins crucial for skin structure and elasticity, are also present in SS. These proteins are essential for maintaining skin firmness and reducing the appearance of fine lines and wrinkles. Their presence in snail mucin has led to its inclusion in many anti‐aging products. This natural substance offers a potent blend of moisturizing, exfoliating, and healing properties as its components work synergistically to promote skin regeneration, boost collagen production, and provide powerful antioxidant effects [23].

Trace amounts of mineral salts (Ca, Mg, Zn, Cu, Fe) and vitamins (A, C, E) are also present in SS [26]. Antimicrobial peptides and copper peptides contribute to their bioactive properties. The exact composition varies depending on the snail species, environmental conditions, and extraction method used. The major components of SS along with their concentrations and detection techniques are listed in Table 1.

**TABLE 1:** Composition of SS including the source species and analytical techniques used to measure the specific compound.

Component	Concentration/presence	Species	Analytical technique	References
Water	90%–99.7%	Various species	Gravimetric analysis	[26, 27]
Proteins	5%–9% of dry weight	*Helix aspersa*	Bradford assay, SDS‐PAGE, and LC–MS/MS	[5, 26, 28]
Glycosaminoglycans	3%–5% of dry weight	*Helix aspersa*	^1^H and ^13^C NMR	[5, 26]
Hyaluronic acid	< 1 mg/g	*Helix aspersa*	ELISA	[4]
Glycolic acid	Up to 4%	*Helix aspersa*	HPLC	[5, 29]
Allantoin	0.3%–0.5%	*Helix aspersa*	HPLC	[29, 30]
Collagen	85 mg/L	*Cornu aspersum*	Fastin‐Elastin Assay	[31, 32, 33]
Elastin	0.099/100 g	*Cornu aspersum*	Fastin‐Elastin Assay	[31, 33]
Glycoprotein	Present	*Achatina fulica*	DEAE‐Toyopearl 650 M ion exchange chromatography	[34]
Copper peptides	Present	*Helix aspersa*	ICP‐MS	[31, 35]
Antimicrobial peptides	Present	Various species	Radial diffusion assay	[36]
Mineral salts (Ca, Mg, Zn, Cu, Fe)	Trace amounts	Various species	ICP‐OES	[17, 35]
Vitamins A, C, E	Trace amounts	Various species	HPLC	[2, 5]
Mucopolysaccharides	1.3%–1.6% of dry weight	*Helix aspersa*	Carbazole assay	[9]
Proteoglycans	Present	Various species	Western blot	[9]
Amino acids profile	Varied	*Helix aspersa*	HPLC‐MS/MS	[37]
Fatty acids profile	Varied	*Cryptomphalus aspersa*	GC–MS	[3]
Molecular weight distribution	10–1000 kDa	*Helix aspersa*	SEC‐MALS	[19]
Secondary structure of proteins	α‐helix, β‐sheet, random coil	*Helix aspersa*	CD spectroscopy	[38]
Functional groups	–OH, –COOH, –NH_2_	Various species	FTIR spectroscopy	[39]
Mucin glycosylation	Complex N‐ and O‐linked glycans	*Achatina fulica*	Lectin microarray	[40]

## 5 Applications of SS

### 5.1 Wound Healing

SS has demonstrated significant potential in wound healing and closure, primarily due to its unique composition of bioactive compounds. The mucus contains several key components that contribute to its wound‐healing properties. Allantoin, present at 0.3%–0.5% in *H. aspersa* slime, promotes cell proliferation and accelerates wound healing. Tsoutsos et al. found that *H. aspersa* extract significantly improved healing in partial‐thickness burns compared to standard treatments [23]. GAGs, comprising 3%–5% of the slime's dry weight, play a crucial role in maintaining skin hydration and supporting the extracellular matrix, both essential for optimal wound healing. HA, a specific GAG found in SS, contributes to this process.

The presence of antimicrobial peptides, as reported by Pitt et al. helps prevent wound infection, further supporting the healing process [36]. Additionally, collagen and elastin in the slime provide a scaffold for new tissue growth and contribute to the strength and elasticity of healed skin. The interlinking property of SS is another significant promoter of wound closure. Deng et al. demonstrated that a hydrogel containing 10% snail mucin accelerated wound closure in a rat model by 23% compared to standard treatment [41]. This effect is likely due to the synergistic action of the slime's components.

The viscous nature of SS also aids in forming a protective barrier over the wound, maintaining a moist environment conducive to faster healing and reduced scar formation. Furthermore, research by Ellijimi et al. showed that SS extract increased the production of growth factors crucial for wound healing in human fibroblasts [6]. Wound healing and closure properties of SS are attributed to its unique combination of allantoin, GAGs, antimicrobial peptides, and structural proteins, which collectively promote cell proliferation, tissue regeneration, and wound protection. The components present in SS and their role in wound healing are listed in Table 2.

**TABLE 2:** Wound healing properties of various components present in SS.

Component	Property	Reference
Allantoin	Promotes cell proliferation and wound healing	[23]
Glycolic Acid	Enhances skin cell turnover	[3]
Glycosaminoglycans	Maintains skin hydration	[5]
Hyaluronic Acid	Contributes to extracellular matrix	[19]
Collagen	Provides scaffold for new tissue	[1]
Elastin	Contributes to skin elasticity	[1]
Antimicrobial Peptides	Prevents wound infection	[36]
Proteoglycans	Modulates inflammatory response	[9]

### 5.2 Treating Skin Conditions

SS has shown promising potential in treating various skin conditions, particularly in burns, diabetic wounds, and dermatitis treatment. Its unique composition of bioactive compounds contributes to its therapeutic effects beyond cosmetic applications.

In burn treatment, SS has demonstrated significant efficacy. A study by Tsoutsos et al. examined the use of *H. aspersa* Müller extract in treating partial‐thickness burns [23]. The researchers found that the snail mucus extract significantly accelerated healing compared to standard treatments. The allantoin content present in the slime is believed to be a key factor in promoting cell proliferation and wound healing in burn cases. For general skin conditions, SS has shown remarkable properties. This effect is attributed to the synergistic action of various components in the slime, including GAGs and HA, which maintain skin hydration and support the extracellular matrix crucial for wound repair.

SS has also shown potential in treating diabetic wounds, a condition often resistant to standard treatments. The antimicrobial properties of the slime, as demonstrated by Pitt et al. help in preventing infection in these vulnerable wounds [36]. Additionally, the presence of collagen and elastin in the slime provides a scaffold for new tissue growth, which is particularly beneficial in chronic wounds associated with diabetes.

In cases of radiation dermatitis, a side effect of radiation therapy in cancer treatment, SS has shown promise. The anti‐inflammatory and hydrating properties of snail mucin suggest potential benefits in soothing and healing irradiated skin. SS has also been investigated for its potential in treating acne and acne scars. The GA content (up to 4% in some species) may help in gentle exfoliation and promote skin cell turnover, potentially reducing the appearance of acne scars [7]. For eczema and psoriasis, the moisturizing and anti‐inflammatory properties of SS offer potential benefits. The high concentration of GAGs and HA in the slime can help maintain skin hydration, while its reported ability to modulate inflammatory responses may help in managing these chronic skin conditions. Snail mucin from *Cryptomphalus aspersa* enhances the growth and movement of keratinocytes and fibroblasts, which are key cell populations for skin repair. This effect increases with higher doses and over time. Additionally, the mucin boosts the production of important adhesion proteins like β1‐integrin, β‐catenin, E‐cadherin, and vinculin, indicating its potential role in promoting scar healing [34].

It is important to note that while these applications show promise, many are still in the research phase. The use of SS in treating skin conditions requires further clinical studies to establish standardized treatments and ensure safety and efficacy across different patient populations. As research progresses, SS may emerge as a valuable natural resource in dermatological treatments, offering alternatives or complementary approaches to conventional therapies for various skin conditions.

### 5.3 Antimicrobial Properties

Antibacterial activities of SS have garnered significant attention in recent years due to their potential applications in medicine and cosmetics. SS has demonstrated efficacy against a wide range of bacterial strains, including both Gram‐positive and Gram‐negative bacteria.

Studies conducted over the past two decades have shown that various snail species produce mucus with antibacterial properties. For instance, *A. fulica* (giant African land snail) mucus has exhibited inhibitory effects against *Staphylococcus aureus* , *Escherichia coli* , and *Pseudomonas aeruginosa* . The mucus from *H. aspersa* has also shown promise, particularly against *Staphylococcus epidermidis* and *Propionibacterium acnes* , making it potentially useful in treating skin infections and acne. The antibacterial activity of snail mucus is attributed to several components, including peptides, glycoproteins, and enzymes. These compounds work through various mechanisms, such as disrupting bacterial cell membranes, inhibiting bacterial growth, and interfering with bacterial communication systems.

Research has demonstrated that the effectiveness of snail mucus varies depending on the snail species, bacterial strain, and concentration of the mucus extract. Notably, some studies have shown that snail mucus can be effective against antibiotic‐resistant bacteria, including methicillin‐resistant *Staphylococcus aureus* (MRSA). Some peptides like mytimacin‐AF isolated from *A. fulica* , and 17 other peptides (Molecular Weight: 3–30 kDa) including proline‐rich and cysteine‐rich peptides isolated from *Cornu aspersum* by Dolashki et al. [42] using MALDI‐TOF/TOF (Matrix‐Assisted Laser Desorption/Ionization equipped with dual Time‐of‐Flight detectors) showed promising antibacterial effects against various species of Gram‐negative and Gram‐positive bacteria. This finding is particularly significant given the growing concern over antibiotic resistance worldwide. A detailed list of SS activities obtained from different snail species against a specific strain of bacteria and mold is listed in Table 3.

**TABLE 3:** Anti‐bacterial/mold activities of SS obtained from different species against particular strains with an experimental minimum inhibitory concentration (MIC).

Snail species	Bacteria/mold	Strain	MIC	Reference
*Achatina fulica*	*Staphylococcus aureus*	ATCC 9080	9.3 mm	[43, 44]
	*Staphylococcus* spp.		15.4 ± 2.04 mm	[1, 45]
	*Streptococcus epidermidis*	ATCC 19606	10.29 mm	[3, 43]
	*Streptococcus* spp.		17.5 ± 2.72 mm	[9, 45]
	*Pseudomonas* spp.		17.1 ± 1.30 mm	[31, 46]
	*Escherichia coli*	PBR 322	21 mm	[35, 42, 47]
	*Vibrio cholerae*		16 mm	[31, 48]
*Archachatina marginata*	*Escherichia coli*		0.098 μg/mL	[45, 49]
	*Salmonella* spp.		0.049 μg/mL	[6, 31]
	*Staphylococcus aureus*		50 μg/mL	[31, 50, 51]
	*Pseudomonas* spp.		50 μg/mL	[31, 36]
	*Escherichia coli*		0.050 μg/mL	[49, 52]
	*Salmonella* spp.		0.098 μg/mL	[31, 53]
	*Staphylococcus aureus*		100 μg/mL	[31, 54]
	*Pseudomonas* spp.		100 μg/mL	[31, 48]
	*Staphylococcus* spp.		17.4 ± 1.20 mm	[45, 55]
	*Pseudomonas* spp.		19.2 ± 1.10 mm	[31, 56]
	*Streptococcus* spp.		18.6 ± 2.14 mm	[31, 57]
	*Staphylococcus* spp.		15.6 ± 1.44 mm	[45, 57]
	*Pseudomonas* spp.		19.8 ± 0.88 mm	[45]
	*Streptococcus* spp.		19.3 ± 1.90 mm	[31]
*Helix aspersa*	*Staphylococcus aureus*	ATCC 25923	> 50 μg/μL	
	*Streptococcus pyogenes*	NCIMB 13285	5.5 mm	[48]
	*Escherichia coli*	ATCC 25922	25 μg/μL	[36]
	*Klebsiella pneumoniae*	NCTC 11228	0 mm	[48]
	*Pseudomonas aeruginosa*	NCTC 8626	11.12 ± 2.57 mm	[36]
		NCTC 10548	11.63 ± 1.52 mm	[31]
		ATCC BAA‐47	25 μg/μL	[31]
	*Candida albicans*	ATCC 10231	> 50 μg/μL	[48]
	*Enterococcus faecalis*	WDCM 00087	23.42 ± 0.68 mm	[58]
	*Staphylococcus epidermidis*	WDCM 00036	24.79 ± 0.21 mm	
	*Staphylococcus aureus*	WDCM 00034	25.04 ± 0.50	
	Methicillin‐resistant *Staphylococcus aureus*	BAA1708	19.58 ± 0.37 mm	
	*Streptococcus pyogenes*	ATCC 19615	23.85 ± 0.18 mm	
	*Bacillus subtilis*	WDCM 00003	22.05 ± 0.28 mm	
	*Bacillus cereus*	WDCM 00001	19.88 ± 0.20 mm	
	*Clostridium perfringens*	WDCM 00007	21.35 ± 0.33 mm	
	*Pseudomonas aeruginosa*	WDCM 00025	20.31 ± 0.53 mm	

	*Enterobacter cloacae*	WDCM 00083	18.60 ± 0.96 mm	
	*Escherichia coli*	WDCM 00013	19.85 ± 0.20 mm	
	*Klebsiella pneumoniae*	WDCM 00097	18.57 ± 0.37 mm	
	*Proteus mirabilis*	WDCM 00023	19.36 ± 0.29 mm	
	*Pseudomonas aeruginosa*	PA‐9	15 μg/μL	[59]
	*Escherichia coli*	EC‐3	20 μg/μL	
	*Staphylococcus aureus*	SA‐17	15 μg/μL	
	*Aspergillus niger*	AN‐05	32 μg/μL	
	*R. stolonifera*	RS	25 μg/μL	
	*Trichoderma harzianum*	TH	25 μg/μL	
*Candida albicans*	CA‐11	20 μg/μL		
*Eremina desertorum*	*Pseudomonas aeruginosa*	PA‐9	7 μg/μL	
	*Escherichia coli*	EC‐3	5 μg/μL	
	*Staphylococcus aureus*	SA‐17	5 μg/μL	
	*Aspergillus niger*	AN‐05	7 μg/μL	
	*R. stolonifera*	RS	10 μg/μL	
	*Trichoderma harzianum*	TH	10 μg/μL	
	*Candida albicans*	CA‐11	12 μg/μL	

While these results are promising, it is important to note that much of the research is still in its early stages. Many studies have been conducted in vitro, and more extensive in vivo research and clinical trials are needed to fully understand the potential of snail mucus as an antibacterial agent in practical applications. Additionally, standardization of extraction methods and quality control measures will be crucial for the development of snail mucus‐based antibacterial products in the future.

### 5.4 Beauty and Cosmetic Uses

SS has gained significant attention in the beauty and cosmetics industry due to its potential anti‐aging, moisturizing, and skin‐regenerating properties. The unique composition of snail mucin, rich in bioactive compounds, has led to its incorporation in various skincare products. Some of the major uses of SS in cosmetics or beauty are explained below.

#### 5.4.1 Anti‐Aging Properties

One of the primary cosmetic uses of SS is in anti‐aging products. The mucin contains glycoproteins, proteoglycans, and GAGs which are known to promote collagen and elastin production. Glycoproteins and peptides present in SS promote proliferation of fibroblasts and production of collagen and elastin which decrease with the passage of time in elderly people thus inhibiting the aging signs [34]. The antioxidants present in SS also contribute to detoxing the oxidation process thus promoting skin's fineness and texture. A study by Fabi et al. demonstrated that a topical gel containing purified snail secretion improved the appearance of fine lines and wrinkles after 12 weeks of use [22]. The presence of allantoin and GA in snail mucin contributes to its skin‐renewing properties, potentially reducing the visible signs of aging [23].

#### 5.4.2 Moisturizing Effects

Snail mucin is highly valued for its moisturizing properties. The high concentration of hyaluronic acid in SS, as reported by Gubitosa et al. helps to maintain skin hydration by attracting and retaining moisture [19]. HA, a natural substance present in SS has the property to hold water 1000 times of its molecular weight. This natural moisturizer can help to improve skin texture and reduce the appearance of dry, and flaky skin. The effect of SS on skin elasticity was evaluated using the Cutometer dualMPA580, showing positive results. This suggests that SS can improve skin elasticity, making it suitable for dermatological applications. In vitro experiments on keratinocytes revealed that SS promotes cellular well‐being, which is essential for maintaining healthy and hydrated skin [58].

#### 5.4.3 Skin Regeneration and Repair

The regenerative properties of snail mucin make it a popular ingredient in products aimed at improving skin texture and reducing scarring. Brieva et al. found that snail secretion filtrate stimulated the proliferation of fibroblasts and the production of extracellular matrix components, suggesting potential benefits for skin regeneration [3]. SS stimulates the proliferation and migration of fibroblasts and keratinocytes, essential for tissue repair and regeneration. It also enhances the expression of genes like TGF‐β1 and VEGF, which are critical for wound healing [59].

#### 5.4.4 Antioxidant Properties

Snail mucin contains antioxidants that can help to protect the skin from free radical damage. These include vitamins A, C and E, as well as antioxidants (e.g., polyphenols) and enzymes (superoxide dismutase—SOD and glutathione S‐transferase—GST) [4]. A study by Mubarak et al. demonstrated the antioxidant properties of snail mucus extract, suggesting its potential in protecting skin cells from oxidative stress [60]. SS has been used to synthesize chitosan and gold‐based nanoparticles, which exhibit significant antioxidant activity. This was demonstrated through DPPH (2,2‐diphenyl‐1‐picrylhydrazyl) and ABTS (2,2′‐azino‐bis(3‐ethylbenzothiazoline‐6‐sulfonic acid)) assays, indicating their potential use in cosmetics as multifunctional ingredients [61].

#### 5.4.5 Anti‐Inflammatory Activities

SS has been shown to possess anti‐inflammatory properties. Research indicates it can help reduce inflammation and promote healing [2]. Specifically, the mucus derived from snails like *H. aspersa* and *A. fulica* contains anti‐inflammatory and antioxidant components that may alleviate conditions such as colon inflammation and enhance overall wound healing [62]. Ricci et al. evidenced that SS reduces the inflammatory events by downregulating the cyclooxygenase‐2 (COX‐2) gene expression in human gingival fibroblasts exposed to an inflammatory stimulus represented by hydrogen peroxide [7]. SS‐based gold nanoparticles significantly reduced LPS‐induced interleukins (IL) levels, such as IL1‐β and IL‐6, in murine macrophages, and completely abrogated the synthesis of inducible Nitric Oxide Synthase [7, 19]. The mucin fraction of *Eremina desertorum* showed antioxidant and anti‐inflammatory effects in experimentally induced intestinal inflammation and testicular damage in mice [63]. SS significantly modulated the expression of inflammatory mediators in canine progenitor epidermal keratinocytes, showing a protective effect on cell viability when stimulated with LPS [64]. SS from *H. aspersa* and *Eremina desertorum* exhibited anti‐inflammatory activities in vitro, including membrane stabilization of human red blood cells, albumin denaturation, and proteinase inhibitory activity [59].

#### 5.4.6 Acne Treatment

Some cosmetic products incorporate snail mucin for its potential in treating acne. The antimicrobial properties of SS, as reported by Pitt et al. may help in managing acne‐causing bacteria [36]. A pilot study demonstrated that a facial cream containing the probiotic strain *Weissella viridescens* UCO‐SMC3, derived from *H. aspersa* mucus, effectively reduced acne lesions in volunteers. This suggests that SS‐based products can be effective in clinical settings for acne treatment [65]. Additionally, the antimicrobial, anti‐inflammatory, skin regeneration, skin hydration, and wound‐healing properties of SS could potentially help to treat acne and reduce acne scarring.

#### 5.4.7 Skin Brightening

Some cosmetic products claim that snail mucin can help in evening out skin tone and reduce hyperpigmentation. This effect is often attributed to the presence of GA, which is known for its gentle exfoliating properties [23]. This effect is further supported by skin regenerative properties of SS.

### 5.5 Emerging Applications

SS, beyond its applications in wound healing, burn treatment, and cosmetics, has shown potential in various other health‐related fields. These applications, while some are still in the early research stages, demonstrate the versatility of this unique biological substance.

#### 5.5.1 Cough Suppressant

SS moisturizes and lubricates the mucous membrane of the respiratory tract and promotes the regeneration of the epithelium. Some studies suggest that preparations derived from snails can decrease morbid activities in pulmonary inflammation, potentially alleviating cough related to these conditions. Due to its emollient and antibacterial properties, SS is considered as an attractive remedy for whooping cough and chronic bronchitis such as tuberculosis [66].

#### 5.5.2 Antiviral Activity

Recent research has explored the antiviral properties of snail mucus. Hemocyanins isolated from the mucus of *Rapana venosa* showed antiviral activity against Herpes Simplex Virus type 1 (HSV‐1) and Human Immunodeficiency Virus type 1 (HIV‐1) [67]. Furthermore, Cilia and Fratini confirmed that slime obtained from *Phyllocaulis boraceiensis* is effective against the single‐stranded Measles virus, and this property is probably due to the presence of polyunsaturated fatty acids specifically hydroxy‐tritriacontapentaenoic acid and hydroxy‐pentatriacontapentaenoic acid [31]. This finding opens up possibilities for SS components in antiviral therapy development.

#### 5.5.3 Dental Applications

The potential use of snail mucin in dental care is an emerging area of interest. A study by Kanthi et al. investigated the effects of snail mucin on dental remineralization. They found that snail mucin enhanced the remineralization of enamel, suggesting its potential use in preventing dental caries [68, 69, 70]. SS has been shown to decrease the number of osteoclasts in rats with periodontitis when administered both orally and topically. This suggests its potential in reducing bone resorption associated with periodontal disease [71].

#### 5.5.4 Osteoarthritis Treatment

The GAGs present in snail mucin, particularly chondroitin sulfate, have been studied for their potential in treating osteoarthritis. While most research has focused on marine snails, the principles could potentially apply to terrestrial snail mucin as well. A review by Zhu et al. highlighted the potential of marine snail‐derived compounds in managing osteoarthritis [72].

#### 5.5.5 Neuroprotective Effects

Some components of SS have shown neuroprotective properties. A study by Guo et al. found that a peptide derived from *A. fulica* mucus exhibited neuroprotective effects in a cellular model of Parkinson's disease [2]. This suggests potential applications in developing treatments for neurodegenerative disorders by acting as an antioxidant against various reactive oxygen species and elevating the level of first‐line enzymatic antioxidants that is, superoxide dismutase (SOD), catalase, reductase, and glutathione peroxidase essential for neuroprotective effects [73, 74, 75, 76, 77].

#### 5.5.6 Gastrointestinal Health

The mucoadhesive properties of snail mucin have been explored for potential applications in gastrointestinal health. Adikwu and Alozie investigated the use of snail mucin as a bioadhesive polymer for drug delivery in the gastrointestinal tract, potentially improving the efficacy of orally administered medications [1, 2]. SS forms an adhesive and protective layer on the internal lining of the gastrointestinal tract and thus prevent the ulcerative effects of acid–base digestive enzymes. SS also contains copper which is proven to have anti‐ulcerative properties [33].

#### 5.5.7 Cancer Research

While still in the very early stages, some studies have investigated the potential anticancer properties of snail mucus components. Matusiewicz et al. reported that lectins isolated from the mucus of *H. aspersa* showed antiproliferative effects on in vitro human colon cancer cells (Caco‐2) [78]. Chien et al. reported that snail mucus enhances chemosensitivity of triple‐negative breast cancer via activation of the Fas pathway [79], likewise, Bo‐Rong and Wei‐Chien reported that snail mucus increased the anti‐cancer activity of anti‐PD‐L1 antibody in melanoma [80]. Rashad et al. stated that *H. aspersa* slime inhibits the growth of IGR‐39 and SK‐MEL‐28 melanoma cells, by increasing expression of the cytokine Tumor Necrosis Factor (TNF‐α), and inhibits the transcription process, by blocking transcription nuclear factor kappa activated B cells (NF‐κB), that in proper regulation has been linked to cancer progression [2]. However, much more research is needed to understand the implications and potential applications in cancer treatment.

#### 5.5.8 Tissue Engineering and Optimized Drug Delivery

The complex composition of snail mucin, rich in growth factors and extracellular matrix components, has attracted interest in the field of tissue engineering. Gubitosa et al. suggested that snail mucus could potentially be used as a bioactive scaffold in tissue engineering applications [19]. Furthermore, the gel‐like nature and interlinking properties make SS an attractive biomaterial candidate for targeted and/or sustained drug delivery with an impactful beneficial effect on gastric mucosa offering a superb gastrointestinal protection.

#### 5.5.9 Ophthalmic Applications

The lubricating and healing properties of snail mucin have led to investigations into its potential use in ophthalmic treatments. While research is limited, there is interest in exploring snail mucin as a component in eye drops for dry eye syndrome or in promoting corneal wound healing [32].

In conclusion, while many of these applications are still in the early research stages, they highlight the diverse potential of SS in various health‐related fields. From antimicrobial and antiviral applications to potential uses in dental care, osteoarthritis treatment, and even neurological and cancer research, snail mucin continues to intrigue researchers with its unique properties.

## 6 Future Prospects

Research is exploring the use of snail mucin as a bio‐adhesive carrier for drug delivery systems. Its unique properties could enhance drug absorption and targeting. A study by Adikwu and Alozie suggested that snail mucin could improve the bioavailability of certain drugs [1].

The complex composition of snail mucin, rich in growth factors and extracellular matrix components, shows potential in tissue engineering. Gubitosa et al. proposed that snail mucus could serve as a bioactive scaffold for tissue regeneration [19].

There's growing interest in the potential of snail mucin as a nutraceutical supplement. Its high protein content and unique bioactive compounds could offer health benefits when consumed as food, though more research is needed in this area [81].

Advanced sensors and IoT technology are being integrated into snail farming. These systems can monitor and control environmental conditions more precisely, optimizing mucin production and snail health [82]. New extraction techniques are being developed to minimize stress on snails. For instance, electrostimulation methods are being explored as a gentler alternative to traditional extraction methods [2, 83]. Research is ongoing into using biotechnology to enhance mucin production in snails or even produce snail mucin‐like substances in laboratory settings without involving live snails [5]. Improvements in chromatography and filtration technologies are enabling more efficient isolation of specific bioactive compounds from snail mucin, potentially leading to more targeted and effective products [6].

## 7 Ethical Considerations

As the use of snail mucin in health and cosmetics has grown, there are more concerns about ethical considerations. The development of “cruelty‐free” Cherasco Muller method for the collection of SS, emphasizes the importance of animal welfare in this area [2, 7].

## 8 Potential for Sustainability

The snail farming industry is exploring ways to utilize all parts of the snail, not just the mucin. This includes using shells for calcium supplements and snail meat for food, moving towards a zero‐waste model [15]. Research is ongoing into developing more sustainable snail farming practices, including optimizing feed formulations to reduce environmental impact and exploring vertical farming techniques to minimize land use [83]. While not a direct use of SS, research into creating synthetic versions of key snail mucin components could reduce reliance on animal‐derived products, potentially offering a more sustainable and scalable alternative [84]. Efforts are being made to establish industry‐wide standards for snail mucin production and quality. This could lead to more sustainable and ethical practices across the industry [23]. Some researchers are exploring how sustainable snail farming could contribute to biodiversity conservation, particularly for endangered snail species [85, 86]. The future of SS in various applications looks promising. Advancements in production technologies, coupled with a growing understanding of its bioactive properties, are opening new possibilities. However, these developments must be balanced with ethical considerations and sustainable practices to ensure the long‐term viability and acceptability of snail mucin‐based products.

## 9 Conclusion

SS, a complex biological substance produced by various species of snails, has emerged as a multifaceted resource with significant potential in diverse fields, particularly in health, cosmetics, and biotechnology. The unique composition of snail mucin, rich in proteins, GAGs, HA, and bioactive compounds, underpins its wide‐ranging applications and future prospects.

In the realm of health and medicine, SS has demonstrated remarkable properties in wound healing, burn treatment, and skin regeneration. Its ability to promote cell proliferation, stimulate collagen production, and provide antimicrobial protection makes it a valuable asset in dermatological treatments. Beyond skincare, emerging research suggests potential applications in areas as diverse as dental care, osteoarthritis treatment, and even neuroprotection, highlighting the versatility of this natural substance.

The cosmetics industry has embraced snail mucin as a key ingredient in anti‐aging and moisturizing products. Its ability to hydrate skin, reduce fine lines, and improve skin texture has led to a surge in snail‐based skincare products. The antioxidant properties of SS further contribute to its appeal in protecting skin from environmental damage.

The extraction and production of SS have evolved significantly, with a growing emphasis on ethical and sustainable practices. From traditional farming methods to advanced biotechnological approaches, the industry is continuously innovating to meet the increasing demand while addressing animal welfare concerns. Non‐invasive extraction techniques and precision farming practices are being developed to minimize stress on snails and optimize mucin production.

Looking to the future, SS holds promise in several emerging applications. Its potential in targeted drug delivery systems, tissue engineering, and as a component in advanced biomaterials could revolutionize certain areas of medical treatment. The exploration of snail mucin in nutraceuticals and its possible role in gastrointestinal health opens up new avenues for research and product development.

SS represents a fascinating intersection of nature and biotechnology. Its diverse applications in health, beauty, and potentially in advanced medical treatments underscore its significance as a valuable biological resource. As research continues to uncover new properties and applications of snail mucin, it is likely to play an increasingly important role in various industries. The future of SS lies in balancing its exploitation with sustainable and ethical practices, ensuring that this remarkable natural substance can continue to benefit human health and well‐being while respecting the creatures that produce it.

However, the expanding use of SS also brings challenges. Ethical considerations regarding snail farming and mucin extraction practices remain at the forefront. The need for standardization in production methods, quality control, and regulatory compliance is crucial as the industry grows. Additionally, the development of synthetic alternatives and sustainable farming practices will be vital in ensuring the long‐term viability and acceptability of snail mucin‐based products.

In conclusion, SS has found a significant place in the beauty and cosmetics industry, primarily due to its potential anti‐aging, moisturizing, and skin‐regenerating properties. While many users and some studies report positive effects, more comprehensive research is needed to fully understand and optimize its benefits in skincare applications.

## Author Contributions

Conceptualization: M.R., S.Z.; methodology: M.R.; software: not applicable; validation: S.Z., A.C.; formal analysis, S.Z.; investigation: M.R.; resources: S.S.; data curation: S.Z.; writing – original draft preparation: M.R.; writing – review and editing: S.Z., A.C.; visualization: A.C., S.S.; supervision: A.C., S.Z.; project administration S.Z.; funding acquisition, A.C., S.Z.

## Conflicts of Interest

The authors declare no conflicts interest.

## Data Availability

Data sharing not applicable to this article as no datasets were generated or analysed during the current study.
